# Systemic Scleroderma—Definition, Clinical Picture and Laboratory Diagnostics

**DOI:** 10.3390/jcm11092299

**Published:** 2022-04-20

**Authors:** Anna Kowalska-Kępczyńska

**Affiliations:** Department of Biochemical Diagnostics, Chair of Laboratory Diagnostics, Medical University of Lublin, 20-081 Lublin, Poland; annakowalskakepczynska@umlub.pl

**Keywords:** systemic scleroderma, connective tissue disease, laboratory diagnostic

## Abstract

(1) Background: Scleroderma (Sc) is a rare connective tissue disease classified as an autoimmune disorder. The pathogenesis of this disease is not fully understood. (2) Methods: This article reviews the literature on systemic scleroderma (SSc). A review of available scientific articles was conducted using the PubMed database with a time range of January 1985 to December 2021. (3) Results and Conclusions: The article is a review of information on epidemiology, criteria for diagnosis, pathogenesis, a variety of clinical pictures and the possibility of laboratory diagnostic in the diagnosis and monitoring of systemic scleroderma.

## 1. Introduction

Scleroderma (Sc) is a rare connective tissue disease classified as an autoimmune disorder. The pathogenesis of this disease is not fully understood. Two types of scleroderma can be distinguished: localized scleroderma (LoSc) and systemic scleroderma (SSc), which can present in two forms: limited and diffuse [1].

## 2. Materials and Methods

This article reviews the literature on systemic scleroderma. A review of available scientific articles was conducted using the PubMed database with a time range of January 1985 to December 2021. The search was conducted using the following keywords: ‘systemic sclerosis’, ‘aetiology’, ‘epidemiology’, ‘manifestations’, ‘classification criteria’, ‘biomarkers’. In total, 203 positions were chosen from the articles found.

## 3. Results and Discussion

### 3.1. Historical Origins of Systemic Scleroderma

The earliest reports of scleroderma date back to 400 BC. In the notes of Hippocrates (460–370 BC) we can find references to a specific “thickening of the skin” in some of his patients [2]. The first detailed description of the disease similar to scleroderma was published by Carlo Curzio in Naples in 1753 [2,3,4]. This Italian doctor from Naples described the symptoms of this disease as the appearance of hard, wood-like skin in various parts of the body of a 17-year-old woman. Beside other symptoms, the patient suffered from skin tension around her mouth and neck. In 1836, a Milanese doctor, Giovambattista Fantonetti, first introduced the word ‘scleroderma’ into medical terminology [5]. Describing his patient’s skin lesions, he described the condition as ‘scleroderma generale’. Its name refers very closely to the symptoms of the disease. This is because the word ‘scleroderma’ comes from two Greek words ‘sclero’ meaning ‘hard’ and ‘derma’ meaning ‘skin’ [3].

In 1862 Maurice Raynaud described in his doctoral thesis, entitled ‘Local Asphyxia and Symmetrical Gangrene of the Extremities’, 25 patients with a series of color changes of hands and feet due to vascular lesions, and 30 years later the systemic form of scleroderma was described with symptoms of pulmonary and/or renal involvement and a significant mortality rate, and thus systemic scleroderma (SSc) ceased to be such a great mystery to the medical world [3,5,6]. In the following years, an increasing number of medical publications addressed both the probable pathophysiology, course and treatment options of systemic scleroderma. However, this disease has not been fully understood, so further study in this field is justified, especially since both the prevalence of scleroderma and the incidence of the disease seem to be increasing especially in the last 50 years [2].

### 3.2. Epidemiology

Epidemiological data on systemic scleroderma are not complete, mainly because it is a rare disease. The most descriptions of this disease focus on the analysis of the specific cases. The European Scleroderma Trials and Research (EUSTAR) group database, which is representative of the general Sc population [7,8], is an excellent source of information in this area.

However, two very interesting systematic reviews and meta-analyses of Sc incidence and prevalence have been published recently (2019 and 2021) [9,10]. These studies involved both a wide time range and the importance of gender or geographical region. Interestingly, the literature analyzed [10,11,12,13] suggests that the incidence of Sc increases at the turn of the year. According to the latest studies [10], the prevalence (i.e., number of affected individuals in the population) of SSc is 17.6 per 100,000 in the population. At the same time, the incidence (in other words, the number of newly diagnosed cases) of SSc averages 1.4 per 100,000 persons. The mean age of patients is 40 years.

SSc, like most autoimmune diseases, is far more common in women than in men. It is accepted that the incidence ratio of SSc worldwide in women and men varies between 3:1 and 8:1 [11], although a study conducted in Tokyo 11 years ago [12] found the ratio in this population to be as high as 14.5:1. Recent reports indicate that the prevalence of SSc worldwide among men is 6.0 per 100,000 persons and among women is 28.0 per 100,000 persons. Similar sex differences are observed in regard to the incidence of SSc, which on average is equal to 0.5 per 100,000 persons worldwide for males and 2.3 per 100,000 persons for females [10]. In addition, it has been shown that although men are less likely to develop SSc, they have a more severe course of the disease and a higher rate of premature death than women [11,12].

Aside from sex, differences in epidemiological data are also influenced by geographical region. It was found that SSc occurs on average per 100,000 inhabitants: in 14.8 Europeans, in 25.9 North Americans, in 24.8 South Americans, in 23.8 Oceania residents and in 6.8 Asians [10]. The lack of estimates currently applies only to the African continent, which is mainly due to insufficient data from more than half of the Sub-Saharan countries [14]. Similarly, incidence rates for SSc show the highest rates of new cases in North America (2.0 per 100,000 persons), Europe (1.6 per 100,000 persons) and South America (1.5 per 100,000 persons). In contrast, lower rates are observed in Oceania (1.0 per 100,000 persons) and Asia (0.9 per 100,000 persons). The lowest incidence is reported for Africa (0.2 per 100,000 persons), but due to the aforementioned data gaps, these values may be underestimated [10].

Although significant advances have been made in understanding the pathogenesis of scleroderma and the availability of treatment is at a much higher level than it was just a decade or so ago, SSc still brings a significant impairment in patients’ quality of life and is still associated with a fairly high mortality rate. Mortality in people with SSc is significantly higher than in the general population (Standardized Mortality Ratio (SMR) = 2.72). It has been shown that 5 years from the time of diagnosis, 74.9% of patients survive, while at 10 years it is only 62.5% [14]. Almost 50% of deaths are due to cardiovascular or pulmonary disorders, but the most common cause of death is lung involvement and development of interstitial lung disease (SSc-ILD) associated with systemic scleroderma (35% of deaths) [14,15].

### 3.3. Definition and Criteria for Diagnosis of the Disease

Scleroderma (Sc) is a rare autoimmune connective tissue disease with a complex pathogenesis. It occurs in two forms—as localized scleroderma (LoSc) and as systemic scleroderma (SSc) [1].

LoSc affects skin lesions and can occur in several forms [1,16,17,18,19,20], the most important of which are:

- Plaque morphea (PMLoSc)—lesions in the form of plaques appear on a limited part of the body (usually the trunk area);
- Generalized (GLoSc)—lesions involve multiple parts of the body (at least two anatomical sites) and are much larger and more diffuse (four or more foci in the size of >3 cm in diameter);
- Linear form (LLoSc)—the lesion resembles a band of thickened skin and usually affects the lower and/or upper limb (most often located along the Blaschko line);
- Linear figure of type en coup de saber (LloSceCS, in. ECDS)—lesions appear on the scalp and resemble the shape of saber scars.

Ssc is the systemic form of scleroderma which is described with symptoms of pulmonary, renal and/or cardiac involvement and a significant mortality rate. It can also appear in various forms [1,15,16]. These include:

- Limited systemic scleroderma (lSSc)—characterized by early onset of Raynaud’s phenomenon, skin involvement limited to the hands, forearms, feet and face, fibrotic lesions in the gastrointestinal tract and lungs, and anti-centromere antibodies (anti-CENP-B, -A, -C or -D);
- Diffuse systemic scleroderma (dSSc)—in the course of which Raynaud’s phenomenon coexists with skin lesions, extending proximally to the elbows and may include the trunk, changes in the gastrointestinal tract, lungs, heart, and kidneys, and the presence of antibodies to topoisomerase I (anti-Scl70) and RNA polymerase III (anti-RNAP III).

Several sets of criteria for the diagnosis of scleroderma have been proposed over the years [21]. Barnet [22] created the first classification of scleroderma already in 1978 on the basis of analyzing 118 cases over a period of 25 years. He distinguished three disease types: type 1, limited to skin lesions on the fingers; type 2, involving skin lesions mainly on the limbs; and type 3 (diffuse) with diffuse skin involvement. Two years later, the American College of Rheumatology (ACR) presented the first standard criteria for the classification of systemic scleroderma, including: a primary criterion of proximal finger skin lesions progressing to skin lesions of the extremities, face, neck, or trunk; and secondary criteria (including sclerodactyly, oral lesions, and bilateral pulmonary fibrosis) [23]. A very interesting proposal for the classification of scleroderma was presented in 1988 by LeRoy [24], who, for the first time, differentiated scleroderma into diffuse (dSSc) and limited (lSSc) forms. However, this classification, which was easy to apply in daily clinical practice, did not allow proper treatment of patients in the early stages of the disease and with small lesions [25]. It was therefore necessary to improve the criteria created, which LeRoy himself undertook, but with the collaboration of Medsger in 2001 [26]. These changes mainly concerned the inclusion of capillaroscopy findings in the classification of scleroderma and the differentiation of the early form of the disease. The division of scleroderma into subclasses also considered the classification proposed by Maricq and Valter in 2004 [27]. However, it has not been accepted due to the considerable complexity of the criteria taken into account. The most recent criteria, valid to date, were established by the American College of Rheumatology (ACR) and the European League Against Rheumatism (EULAR) in 2013 [28]. At the initiative of the international group EUSTAR (European Scleroderma Trials and Research), research was also carried out to establish a diagnostic algorithm allowing for early diagnosis of systemic scleroderma. For this purpose, a prospective study called VEDOSS (very early diagnosis of systemic sclerosis) was designed. It was a longitudinal registry study conducted in 42 European scleroderma trial and research group centers, which were located in 20 countries around the world. Patients with Raynaud’s phenomenon were enrolled in the project and monitored according to the four VEDOSS criteria (presence of antinuclear antibodies (ANA), oedema of the fingers, autoantibodies specific for systemic sclerosis and abnormal capillaroscopy of nail folds). Assessment of the presence of antinuclear antibodies (ANA) has been recognized as a significant aspect of identifying patients at risk of developing systemic scleroderma. Their presence and one or two of the other VEDOSS criteria in patients with Raynaud’s phenomenon puts them at high risk of developing systemic sclerosis, increasing additionally over time [29,30,31]. Thanks to their introduction and application, it is possible to diagnose and monitor systemic scleroderma in a much more systematic way (as it is supported by the use of scoring), which, however, does not diminish the great role of the work of the other scientists mentioned. Comparisons of the most commonly used diagnostic criteria for scleroderma, i.e., LeRoy 1988, LeRoy/Medsger 2001, and ACR/EULAR 2013, were made in Table 1.

### 3.4. Aetiology and Pathogenesis

The etiology of systemic scleroderma has not been fully understood yet. However, it is generally believed that genetic and environmental factors are the main contributors to its development [1]. In the case of genetic relationships, it is not yet possible to identify a single ‘culprit’. Current literature reports that most of the identified genes that could account for the propensity to develop SSc are also linked to other autoimmune diseases (so-called ‘shared autoimmunity’). However, the association of the HLA loci DRB1*1104, DQA1*0501 and DQB1*0301 and PTPN22, NLRP1, STAT4, and IRF5 with SSc has been demonstrated to date [32,33,34,35]. A possible role for miR-21 (microRNA 21) and miR-29 has also been described [36,37]. Among environmental factors proven to favor the development of SSc, the most important ones are infectious agents [38]: cytomegalovirus (CMV) [39], Epstein–Barr virus (EBV) [40], and parvovirus B19 [41]. It has also been shown that exposure to pollutants and certain chemicals can also initiate pathological changes associated with SSc. These include exposure to: silica dust [42], organic solvents [43], toluene, xylene, trichloroethylene [44], and polyvinyl chloride [45].

Systemic scleroderma combines vascular, inflammatory, immunological, and blood coagulation disorders in its course. A damaging factor, either genetic or environmental, can lead to dysfunction of vascular endothelial cells, causing them to over-activate [46]. It is generally known that the endothelium secretes numerous vasomotor substances, influencing coagulation and fibrinolysis, involved in the regulation of inflammatory processes, interaction between the vessel wall and leukocytes and platelets, and substances affecting the permeability of the vessel wall [47]. As a result of endothelial cell activation by a damaging factor, an imbalance between these processes can occur. This results in an impaired synthesis of modulators of vascular wall tone due to excessive endothelin synthesis and reduced synthesis of nitric oxide (NO) and prostacyclins. Increased expression of adhesion molecules (ICAM, VCAM) and synthesis of chemokines, cytokines, and growth factors are also observed [46]. These activities definitely promote the recruitment of cytokines and inflammatory cells.

The cellular and molecular interactions and changes that occur during SSc are quite poorly understood. However, it is known that inflammatory cells involved in the ongoing process are: monocytes, macrophages polarizing mainly towards M2 cells, dendritic cells, mast cells, CD4+ lymphocytes (mainly Th2 cells), and activated B lymphocytes. They synthesize interleukins (IL-1, IL-4, IL-6, IL-10, IL-13), growth factors (TGFβ, PDGF, CTGF, VEGF), type I interferons (IFN-α, IFN-β), autoantibodies or even enzymes (arginase-1) causing excessive proliferation of vascular inner membrane cells and smooth muscle cells and activate fibroblasts, which in a completely disorganized way start to synthesize the extracellular matrix (ECM) [48,49,50,51,52,53]. Persistence of this condition leads to accumulation of reactive oxygen species, hypoxia and synthesis of growth factors, leading to vascular remodeling and tissue fibrosis. The deposits of collagen, but also of hyaluronic acid, glycosaminoglycans or fibronectin, form a thick and rigid connective tissue that destroys the original architecture and disrupts tissue function [46].

### 3.5. Clinical Picture

The most characteristic feature of SSc is the presence of skin lesions, which is found in almost all patients. Even the name of the disease itself comes from the typical loss of elasticity of the skin, accompanied by a strong feeling of tension, which over time develops into thickening and induration. The severity of the lesions and their location can of course vary from person to person. Based on these differences, as mentioned earlier, a limited form of the disease (lSSc) and a diffuse form (dSSc) can be distinguished [1,54].

Abigun and colleagues [1] in their work on systemic scleroderma provide an excellent account of the skin changes that can be observed in the course of this disease. They distinguish three phases in the development of cutaneous manifestations. The first of these, the so-called ‘puffy finger phase’, is associated with worsening of inflammation within the individual skin layers, resulting in non-pitting oedema of the fingers and hands, itching, and pain. The accompanying pressure on internal structures often leads to compression neuropathy and loss of skin appendages. Very typical of this stage is the occurrence of Raynaud’s phenomenon (periodic ischemia of the fingers caused by cold or emotional stress). In the second phase of skin lesions in the course of SSc, the so-called ‘prolonged fibrotic phase’, there is a gradual hardening of the skin of the fingers (sclerodactyly), starting from the metacarpophalangeal joints. These thickenings may be accompanied by ulcerations, scarring, and bacterial superinfections of the fingertips, which not only significantly impair the patients’ quality of life, but may also result in amputation. Skin changes at this stage also begin to affect the facial skin. Numerous telangiectasias (vascular spider veins), deformity of the nose resembling a bird’s beak, and an acquired microstomia (abnormally small mouth) along with radial furrows around the mouth are observed. Sclerosis and thickening together with the aforementioned lesions distort the patient’s face often collectively forming the so-called ‘mask image’ [54,55]. ‘Skin-softening phase’ is the last and rare stage of skin lesion development in SSc. The surface layers of the skin may soften over time, returning to their original state. However, this change does not affect the subcutaneous layer, which will already be permanently characterized by the presence of fibrous lesions [1].

A very common cutaneous manifestation of late SSc is the deposition of insoluble calcium in the skin and subcutaneous tissues, the so-called skin calcifications. The available literature suggests that it is caused by blood supply disorders in the skin and subcutaneous tissue present in SSc. The presence of calcinosis correlates with symptoms such as finger ulcers and acroosteolysis in most patients [56,57,58]. Calcinosis occurs both in patient populations with the presence of anti-centromere antibodies (ACA), anti RNA polymerase III antibodies, and in the presence of anti Scl-70 antibodies [59,60,61]. It occurs in both lSSc and dSSc patients [62]. It was confirmed by Valenzuela’s team in 2020 [56], showing that the type of autoantibodies and type of SSc is not important in the prevalence of this symptom in patients, and that ethnic and geographical factors may be determinants [56].

Telangiectasias, already mentioned when describing facial skin lesions and included in the 2013 classification criteria, are also a common skin lesion and can also affect the hands, mucous membranes, and even the trunk [1]. These changes are mainly due to the dilatation of blood vessels, specifically postcapillary venules, within the patient’s skin [63]. There are reports on the association of telangiectasia with the development of pulmonary hypertension [64,65,66].

Skin lesions appearing in patients with SSc correlate with structural abnormalities observed in the microvascular area. These are assessed non-invasively using the nailfold capillaroscopy technique, which allows the number and morphology of capillaries and the presence of subcutaneous hemorrhages to be checked. Over the years of studies, the so-called ‘scleroderma pattern’ has been established, which consists of the presence of giant capillaries (with an apical diameter ≥ 50 μm) or the presence of abnormal vessel morphology with a reduced number of capillaries [67]. X-ray and ultrasound are also used to analyze skin lesions. X-rays allow acroosteolysis (16%), calcifications (46%), and soft tissue thinning to be captured, while ultrasound enables the thickness and echogenicity of the skin to be assessed and edematous lesions to be distinguished from fibrous lesions [68]. Besides, ultrasound shear wave elastography (US-SWE) is proving to be an interesting tool, increasingly used to assess the number of fibrous changes in the skin by evaluating its tension [69]. Blood flow in the subcutaneous tissues can also be assessed using color Doppler ultrasound. Increased flow may be suspected, among other things, by increased proliferation of the synovial membrane of the joints [70]. These pathologies are also visible in magnetic resonance imaging (MRI) studies [71]. An interesting diagnostic solution may also be the use of artificial intelligence with deep convolutional neural networks (CNNs). The use of CNN can support the diagnosis of skin lesions in the course of systemic scleroderma by using algorithms that analyze photos of the affected areas of the skin [72,73].

One of the most common (about 90% of patients) clinical presentations of SSc, in addition to cutaneous manifestations, is involvement of the gastrointestinal tract with lesions [74,75]. They can affect the entire length of the gastrointestinal tract (GIT), starting in the mouth and ending in the anus. On average, 30–70% of symptoms are related to oral lesions, 80–90%, and therefore most of them, to esophageal dysphagia. Changes in motility and patency of the small intestine (60–80%) and the large intestine (20–50%) and problems with defecation associated with rectal lesions are also fairly common findings [76,77,78]. Gastrointestinal involvement may be due to vascular lesions that directly affect GIT motility by the presence of ischemia. It leads to damage to the innervation of, among others, the intestinal wall and progressive fibrosis of the muscle tissue up to complete atrophy [79]. Furthermore, gastrointestinal symptoms are also due to mucosal damage by significant infiltration by T lymphocytes, resulting from immunological instability in SSc [80].

The manifestations of upper gastrointestinal tract involvement by SSc include microstomia, xerostomia (decreased saliva secretion), taste disorders resulting from inflammation of the oral mucosa, periodontium and taste buds [81], dysphagia, lower esophageal sphincter (LOS) abnormalities, gastroesophageal reflux disease (GERD), Barrett’s esophagus, and even esophageal adenocarcinoma [82]. Esophageal changes, in the form of abnormal fluid or air filling, can be visualized on computed tomography (CT) [83]. Gastric antral vascular ectasia (GAVE) is very characteristic of SSc with GIT involvement. These lesions, leading to acute and chronic gastrointestinal bleeding and related complications, resemble red bands arranged in the prepyloric part of the stomach along its folds, reaching up to the pylorus, the so-called ectasias, and are often referred to by this characteristic image as ‘watermelon stomach’ [84]. Within the small bowel, patients with SSc may develop chronic intestinal pseudo-obstruction (CIPO). During its course, as a result of intestinal motility disorders, patients experience chronic nausea, abdominal pain, flatulence, and frequent constipation [85]. Quite rarely, the development of pneumatosis cystoids intestinalis (PCI) can also be observed, which is characterized by the presence of numerous cysts in the intestinal wall, filled with gas. Most of these pathologies can be demonstrated by endoscopic examination [86]. Small intestinal bacterial overgrowth syndrome (SIBO), which develops due to an increased number of bacteria and manifests as flatulence, abdominal pain and diarrhea, can also lead to digestive and absorption disorders in SSc [87].

Respiratory abnormalities in SSc primarily involve systemic scleroderma-associated interstitial lung disease (SSc-ILD) [88] and the development of pulmonary arterial hypertension (PAH), resulting from remodeling of small pulmonary vessels [46]. There are only sporadic reports on the development of airway obstruction in SSc. Rather, the cases indicate the importance of smoking in pulmonary changes becoming more severe in scleroderma [89,90]. A few patients developed cylindrical bronchiectasis, a progressive, irreversible dilatation of the bronchial tree [91]. There are also reports of changes in pulmonary capillaries leading to diffuse alveolar hemorrhage (DAH) [92,93] and the development of pleuritis associated with lymphocytic effusion [94,95]. However, SSc-ILD (35% of SSc-related deaths) and PAH (responsible for 26% of deaths) are now the leading causes of death in systemic scleroderma.

SSc-ILD can develop in SSc patients ranging from limited non-progressive lung involvement to severe respiratory failure resulting in a patient’s death [88,96]. It has been found to be more common in patients with diffuse forms of SSc [97]. The presence of antibodies against topoisomerase I (85%), correlating with SSc-ILD activity, is very characteristic for these individuals [98,99,100]. The opposite findings were made for anti-centromere antibodies, the presence of which is associated with a low incidence of SSc-ILD [101]. Genetic factors such as a link of CTGF to functional polymorphism [102], a link to IL6 gene [103], rs763361 single nucleotide polymorphism (SNP) in the CD226 gene [104], or genetic polymorphisms of IL-1α and IL-1β genes [105,106,107] have also been observed to be of high importance in the development of SSc-ILD. The pathogenesis of pulmonary lesions in SSc itself is not yet fully understood. Pulmonary manifestations are assumed to be related to abnormal interactions between endothelial cells, inflammatory cell response (mainly monocytes and lymphocytes) and fibroblast activation, leading to excessive production of extracellular matrix. This abnormal state of cellular hyperreactivity is further complicated by a state of respiratory tissue hypoxia and vascular changes [108].

X-ray imaging is of less importance for the diagnosis of pulmonary lesions in SSc, due to the fact that pulmonary fibrosis is only visible in advanced stages of the lesions using this method [109]. Ultrasonography can be helpful in imaging areas of irregular pleural thickening, but the primary method for diagnosing pulmonary manifestations of SSc is high-resolution computed tomography (HRCT) [68].

Systemic sclerosis associated pulmonary arterial hypertension (SSc-PAH) is considered to be a condition in which the mean pulmonary artery pressure (PAP), measured in a patient with SSc using right heart catheterization (RHC), is greater than 20 mmHg in the supine position and at rest. In addition, measurement of pulmonary capillary wedge pressure (PCWP) indicates values less than or equal to 15 mmHg and pulmonary vascular resistance (PVR) ≥ 3 Wood units [110]. PAH is estimated to occur in SSc patients much less frequently than ILD, with 7–12% of cases [111]. The pathogenesis also of this pulmonary lesion in SSc is not entirely clear. However, pulmonary artery stenosis is suspected to be caused by fibrosis due to vascular endothelial damage and dysregulated inflammatory response and angiogenesis [112]. The gold standard for the diagnosis of PAH is pulmonary arteriography (right heart catheterization) [68].

The first description of the cardiac manifestation of systemic scleroderma presented fibrous lesions in the coronary arteries, pericardium and myocardium [113]. It is now thought that approximately 27% of deaths in patients with SSc are due to heart diseases [114]. These most commonly include myocarditis, development of coronary artery disease, myocardial fibrosis, conduction system abnormalities, valvular regurgitation, heart failure, or pericardial and/or endocardial disease [115,116]. There are increasing reports of more frequent cardiovascular lesions in the course of dSSc and with the presence of antibodies against topoisomerase I [116,117,118,119].

Heart lesions in SSc develop very insidiously. In the initial phase, cardiac functional and vasodiastolic abnormalities do not cause any clinical symptoms and are mostly reversible [120]. However, these changes deepen over time. The structure of the coronary arterioles and small arteries begins to remodel, leading to reduced flow in the coronary circulation, affecting the myocardium. As a result of microcirculatory disturbances, local ischemia begins to occur and this in turn can lead to worsening myocardial fibrosis [116,121]. Myocardial fibrosis sometimes leads to impaired myocardial relaxation and hypertrophy of both heart ventricles, leading to advanced diastolic and systolic cardiac dysfunction and arrhythmias [122]. Changes in the structure of the coronary vessels, impaired coronary circulation, and progressive fibrosis are not the only causes of arrhythmias in SSc. As a result of the ongoing inflammatory response, myocardial oedema also frequently occurs here [123], which also results in the development of arrhythmias in the long term. Unfortunately, some of the medications used to treat SSc can also cause heart rhythm disorders to become more severe. Such substances include methotrexate, which is often used in the treatment of lSSc [115,124,125]. Less common cardiac manifestations include: pericardial disease, which fortunately for patients is usually benign, left or right ventricular dysfunction and developing heart failure, which, although infrequent, often lead to a patient’s death [122,126]. Symptoms of these changes are increasing dyspnea and oedema. The pathogenesis of pulmonary lesions in SSc itself is not yet fully clarified. There are still new reports on the subject, but vascular lesions resulting from vascular endothelial damage, leading to progressive fibrosis, are considered the most likely source of the resulting lesions [115,127]. For the assessment of cardiac morphology and function, very useful imaging modalities are CT (computed tomography), which can visualize pericardial fibrosis and effusion, and MRI, whose techniques such as ‘wall tagging’ and PC-MRI (phase-contrast magnetic resonance imaging) are also useful for assessing fibrosis and diastolic heart failure [68,128,129,130].

Cardiac and pulmonary changes in SSc are very often accompanied by pathologies in the urinary system. Interestingly, kidney lesions develop much more rapidly than, for example, pulmonary lesions in SSc. This is mainly due to the higher blood pressure in the renal circulation compared to the vessels of the respiratory system [131]. Vascular fibrosis within the kidney very quickly leads to damage to the renal glomeruli and impaired glomerular filtration. Unfortunately, the clinical manifestations caused by these pathologies only become apparent when most of the renal tissue is destroyed. In about 50% of patients with SSc, laboratory tests show abnormalities characteristic of impaired urinary function, such as proteinuria, decreased GFR, increased creatinine levels, developing hypertension, or the so-called scleroderma renal crisis (SRC) [132,133,134]. SRC is characterized by malignant hypertension and rapidly progressive renal failure. Patients with SRC are characterized by high blood pressure, decreased estimated glomerular filtration rate (eGFR), schizocytosis in a blood smear (>1%) and symptoms of hemolysis, hematuria, or proteinuria [135]. It is a life-threatening condition and the prognosis for the patient is poor. SRC is caused by damage to the vascular endothelium. The basic process is the intimal proliferation of the interlobular and arcuate arteries of the kidneys. This proliferation leads to a reduction in renal perfusion and glomerular hyperplasia and renin secretion. The classical activation of the complement system, the renin–angiotensin system, and some factors that promote and maintain inflammation are believed to be involved. As a result, acute arterial hypertension and acute renal dysfunction develop [136,137,138]. A renal biopsy from patients with SRC showed active and chronic thrombotic microangiopathy (TMA), which is a pathological condition characterized by destructive thrombocytopenia and microangiopathic hemolytic anemia [139]. Impairment of vascular endothelial function in SRC results in an increase the number of unusually large von Willebrand factor multimers (UL-VWFM) over the amount that can be cleaved efficiently by ADAMTS13 (a disintegrin and metalloproteinase with thrombospondin type 1 motif, 13). UL-VWFM remain uncleaved in the circulation, which causes the formation of platelet clots [136,137,140,141]. The abnormalities found in the renal arteries are caused by intimal proliferation and vascular remodeling (an image of “onion bulbs” in the arteries). The lumen of the arteries may be completely obstructed, which leads to a reduction in glomerular filtration [136,137,138]. Progressive renal vascular and renal tissue fibrosis accounts for the significantly worse prognosis in patients with SSc, as confirmed by the available literature [142,143,144].

The most common images for SSc are presented in Table 2.

### 3.6. Laboratory Diagnostic

Laboratory tests used for the diagnosis of systemic scleroderma are still of limited relevance at present and are mainly based on the determination of autoantibodies included in the ACR/EULAR 2013 criteria, i.e., anti-centromere antibodies, topoisomerase I antibodies and RNA polymerase III antibodies, which show the highest specificity for SSc [145]. However, it is important to remember that in addition to being able to make a diagnosis using these determinations, their results can also be extremely helpful in linking the course of SSc to specific clinical symptoms and disease progression over time.

One of the laboratory tests mentioned is the detection of the presence of antibodies against topoisomerase I (anti-Scl 70) in the serum of patients. Demonstration of these autoantibodies in a patient is often associated with significant skin involvement by fibrotic lesions. In addition, patients are more likely to develop severe internal organ lesions, especially SSc-ILD [145,146,147]. The presence of anti-RNA polymerase III (anti-RNAP III) antibodies in the patient’s serum also indicates an increased risk of developing dSSc. Skin lesions in this group of patients grow significantly faster and renal complications—gastric vascular ectasias and neoplastic lesions are more common in the disease [24,145,148,149,150,151,152,153]. The presence of anti-centromere antibodies (anti-CENP-B, -A, -C or -D) in the patient’s serum is definitely more beneficial from a clinician’s perspective. These autoantibodies are associated with a moderate degree of skin fibrosis. Organ involvement in SSc is slower in these patients and is usually limited to progressive involvement of finger vessels and development of PAH. The presence of anti-CENP antibodies has also been shown to correlate negatively with the occurrence of neoplastic lesions in patients with SSc [145,148,149,154,155]. Of course, many other autoantibodies are also detected in SSc. The significance of all of them has yet to be discovered. Studies in this area are still ongoing. Antibodies found in SSc that can be used to predict disease course include anti-U11/U12 RNP antibodies (anti-RNPC3), which have a proven association with the risk of severe GIT manifestations and are associated with a higher risk of neoplastic lesions [156,157]. The organ manifestations of SSc also correlate with the presence of anti-fibrillarin antibodies (anti-U3 RNP), the presence of which may indicate a higher risk of developing PAH and other cardiac complications [145,158]. The development of PAH is also more common in patients diagnosed with serum anti-Th/To antibodies. These antibodies may also increase the risk of SSc-ILD [159,160]. Many of the autoantibodies detected in SSc may also indicate a stronger stimulation of fibroblasts and endothelial cells in SSc, leading to more rapidly progressive fibrotic changes. These antibodies include, for example, antibodies against endothelial cells [161], antibodies against the PDGF receptor and against endothelin receptors [20]. An interesting study was also published by Shah’s team [162], which showed an association of the presence of antibodies against the large RNA subunit Pol I (RPA194) with a reduced risk of cancer [149]. It is worth highlighting that an increased risk of tumor (involving mainly breast and lung) is observed in SSc, as already mentioned above. To facilitate the use of the correlation of the presence of these autoantibodies with increased or decreased risk of carcinogenesis, Shah and colleagues proposed a detailed screening algorithm for SSc patients [163,164]. Antineutrophil cytoplasmic antibodies (ANCA; 11.2%—anti-MPO, 13.8%—anti-PR3) are also found in patients with SSc [165]. These patients are more likely to develop SSc-ILD along with other pulmonary and renal complications, and have increased mortality [166,167].

The most frequently detected antibodies in the diagnosis of systemic sclerosis are presented in Table 3.

In addition to the assessment of the presence and titer of autoantibodies in the serum of patients, this biological material can also be used to assess the management of organ lesions. A lipid panel (total cholesterol, HDL-cholesterol, LDL-cholesterol, triglycerides), liver (e.g., ALT, AST, GGTP), renal (e.g., eGFR, creatinine, urea, sodium, potassium), and cardiac (e.g., NT-pBNP, BNP, troponin T/I) function parameters are routinely determined in patients with SSc. However, new indicators are still being searched for, which would allow rapid and specific detection of organ changes and consequently the implementation of appropriate treatment. Among the biochemical markers used to monitor the course of SSc, the traditional inflammatory marker C-reactive protein (CRP) cannot be overlooked. Elevated concentrations of this protein significantly correlate in many clinical studies with the severity of skin lesions as assessed by the Rodnan Scale (mRSS) and with a decrease in forced vital capacity (FVC) in SSc-ILD, and are thus associated with a poorer therapeutic response and higher mortality rate than in patients with CRP < 8 mg/L [145,165,168,169,170,171]. The parameters determined in serum are much less frequent: IL-6, whose increase is characteristic of significant skin involvement and the development of SSc-ILD [145,172]; TGF-β, which is suspected to enhance the development of fibrotic lesions within internal organs and skin [173]; CTGF, high levels of which correlate with the extent of skin lesions and severity of SSc-ILD [174]; soluble sCD163, significantly elevated in serum in patients with severe PAH and SSc-ILD [145]; sonic hedgehog homolog (SHH) signaling pathway activity, the increase of which is significant in severe fibrotic skin lesions [175,176]; levels of KL-6 (Krebs von den Lungen) glycoprotein, produced by type II pneumocytes and a predictor of long-term development of end-stage lung disease [165,177,178]; or CCL18/PARC chemokine levels, which correlate with the severity of SSC-ILD [165,179]. Matsuda et al., in their studies showed that the increase in the severity of skin lesions assessed using the Rodnan Skin Score (mRSS) is very strongly associated with the higher incidence of interstitial lung disease (*p* < 0.05), restrictive impairment (*p* < 0.01), and diffusion impairment (*p* < 0.05) in the lungs [180]. In addition, it has been shown that the modified Rodnan Skin Score (mRSS), which involves palpation of the skin of a patient with SSc across 17 anatomical areas within which skin thickness is assessed [181], correlates perfectly with the results of the ELF (enhanced liver fibrosis) test for non-invasive diagnosis and assessment of hepatic cirrhosis progression. This test involves assessing the patient’s serum levels of hyaluronic acid (HA), tissue inhibitor of metalloproteinase 1 (TIMP-1) and procollagen type III amino-terminal fragment (PIIINP) [182]. Increases in concentrations of these parameters also indicate a more severe course of SSc [165].

Gene expression determination is a much less frequently performed laboratory test. However, it is worth mentioning that the association of high expression of five genes (CD14, IL13RA1, SERPINE1, OSMR, and CTGF) with the presence of severe fibrotic skin lesions has been demonstrated [183]. There are also reports of the effect of increased expression of inflammatory genes in the skin, genes of plasma cells in the skin and those responsible for the senescence-associated secretory phenotype (SASP) on the improved response of patients with SSc to the applied treatment [165,184,185,186]. On the other hand, there are reports demonstrating that, for example, parvovirus B19 (B19V) infection, which acts to increase SASP phenotype expression, may represent a novel pathogenic mechanism for skin fibrosis [186]. This therefore demonstrates the need for further research in this area.

### 3.7. Treatment

Treatment of SSc is not standardized. However, it is important that the treatment of patients with systemic sclerosis should be comprehensive and includes both patient and family education, pharmacological treatment and rehabilitation. The therapeutic management should always be determined individually, taking into account the form and duration of the disease, the occurrence of organ complications, as well as the benefits and risks of treatment. Recommendations for the treatment of specific organ complications and interdisciplinary care with the participation of specialists in particular fields of medicine are of great importance here [187,188].

Mycophenolate mofetil (MMF) and cyclophosphamide (CYC) are often considered the first line therapies for the treatment of SSc. The EULAR guidelines for the treatment of SSc [189] include methotrexate also as the first-line treatment for cutaneous sclerosis in SSc. With regard to skin lesions in the course of SSc, immunosuppressive treatment with oral cyclophosphamide (CYC) may significantly reduce the extent of cutaneous sclerosis [190]. Moreover, treatment of CYC leads to significant improvement in patients with ILD (interstitial lung disease) [191]. Treatment with mycophenolate mofetil (MMF) also leads to the improvement in skin sclerosis [192,193]. In patients with ILD, treatment with MMF for 24 months led to significant improvement. Importantly, MMF is better tolerated and has a better safety profile compared to CYC [192].

Hematopoietic stem cell transplantation (HSCT) is usually reserved for patients with rapidly progressive cutaneous sclerosis and organ involvement without significant cardiovascular involvement who are refractory to immunosuppressive therapy. HSCT inhibits the progression of organ changes, reduces cutaneous sclerosis, as well as potentially ameliorating ILD in selected patients with dcSSc [194,195].

However, the most characteristic feature of systemic scleroderma treatment is organ therapy, the aim of which is to protect a specific organ, initiate the treatment of pathological changes as early as possible, and individualize therapeutic treatment.

In this regard, it is worth mentioning that the typical treatment of Reynaud’s phenomenon as well as necrosis and ulcers is the use of calcium channel blockers from the dihydroxypyridine group, phosphodiesterase type 5 inhibitors, fluoxetine, or iloprost. Treatment of patients with SRC requires introduction of ACEI (angiotensin-converting-enzyme inhibitors) to treatment, and treatment of ILD patients with immunosuppressive treatment (CYC or MMF), also with administration of glucocorticoids but with caution and sparingly. The progression of lung disease is also slowed down by anti-fibrotic pirfenidone and nintedanib, which were approved by the FDA for the treatment of SSc-ILD [196]. Moreover, particularly in patients with early dcSSc with inflammatory features in lungs, a hope may be the monoclonal antibody against the interleukin (IL)-6 receptor—tocilizumab [197]. In patients with pulmonary arterial hypertension (PAH) in SSc, the standard of care was monotherapy with prostacyclins, phosphodiesterase inhibitors, and endothelin receptor antagonists, but recent literature suggest that combining tadalafil and ambrisentan is also a promising therapeutic strategy in PAH [198].

An observational study demonstrated that combining rituximab (the B-cell depleting agent) with MMF is safer and leads to significant improvements in mRSS [199]. Additionally, rituximab may possess disease-modifying effects on ILD in SSc and led to a greater improvement in mRSS compared with CYC [200]. This suggest that this agent may play a role in the management of patients with skin and lung disease who fail to respond to conventional immunosuppressive therapies [188].

There are high hopes for research on biological DMARDs (disease-modifying anti-rheumatic drugs) and small-molecule synthetic drugs. Therapies towards inflammatory cytokines (rituximab, tocilizumab, abatacept) and strategies based on autoantibodies status and skin gene profiling may provide new answers and open new avenues in treatment of SSc [201,202,203].

## 4. Conclusions

Systemic scleroderma is a disease with ‘many faces’. The pathomechanism of the observed changes, both cutaneous and organ-related, and the utility of many of the potential biomarkers, are not yet fully understood, despite numerous discoveries in this field in recent years. Further studies are recommended to first understand the mechanisms of development of this disease and then to improve the diagnosis, monitoring, and treatment of patients with SSc.

## Figures and Tables

**Table 1:** Comparison of the selected diagnostic criteria for systemic scleroderma.

	LeRoy 1988 [24]	LeRoy/Medsger 2001 [26]	ACR/EULAR 2013 [28]
**Characteristic of the criteria**	Differentiation between SSc and lSSc.	Consideration of capillaroscopy findings and early SSc form.	Introduction of scoring.
**Clinical criteria**	dSSc: - Short interval (<1 year) between onset of Raynaud’s phenomenon and development of skin lesions; - Peripheral skin involvement and trunk skin lesions; - Tendon involvement; - Changes in the lungs, kidneys, gastrointestinal tract and heart muscle; - Capillary abnormalities of the nail shaft. lSSc: - Long interval (>1 year) between onset of Raynaud’s phenomenon and development of skin lesions; - Limited skin lesions; - Late development of pulmonary hypertension, calcinosis, and telangiectasia; - Dilated capillaries visible in the nail folds.	early SSc: - Raynaud’s phenomenon documented as: (a) pallor; (b) cyanosis; (c) blushing; - Direct measurement of skin response to low temperature: (a) Abnormal findings on widefield NFC; (b) Abnormal Nielsen test result or equivalent; - Capillary abnormalities of the nail shaft typical for scleroderma. dSSc: - Criteria as for the early form and proximal skin lesions. lSSc: - Criteria as for the early form and distal skin lesions	1. Skin induration of both hands proximal to the metacarpophalangeal joints (9 points); 2. Scleroderma of the fingers: swelling of the whole fingers (2 points), sclerodactyly (4 points); 3. Damage to the fingertips: fingertip ulcers (2 points), scars on the fingertips (so-called thimble-like scars) (3 points); 4. Telangiectasias (2 points); 5. Capillary abnormalities of the nail shaft typical for scleroderma (2 points); 6. Pulmonary arterial hypertension and/or interstitial lung disease (2 points); 7. Raynaud’s phenomenon (3 points).
**Laboratory criteria**	dSSc: - Presence of autoantibodies against topoisomerase I (anti-Scl 70). lSSc: - The presence of anti-centromere autoantibodies (anti-CENP-B, -A, -C, or -D).	Early SSc: - Presence of autoantibodies against: (a) centromeres; (b) topoisomerase I; (c) fibrillarin; (d) fibrillin; (e) PM-Scl; (f) RNA polymerase I or III at a ratio of 1:100 or higher.	Autoantibodies specific for systemic scleroderma (max 3 points): - Anti-centromere (anti-CENP-B, -A, -C, or -D); - Against topoisomerase I (anti-Scl 70); - Against RNA polymerase III (anti-RNAP III).
**Interpretation**	dSSc or lSSc can be diagnosed by the presence of typical lesions.	dSSc, lSSc, and early onset can be diagnosed by the presence of typical lesions.	Systemic sclerosis can be diagnosed when the total score is ≥9.

ACR/EULAR—American College of Rheumatology/European League Against Rheumatism, lSSc—limited systemic scleroderma, dSSc—diffuse systemic scleroderma, SSc—systemic scleroderma, NFC—nailfold capillaroscopy, CENP—centromere, anti-PM-Scl—antibodies associated with polymyositis (PM)/systemic scleroderma (SSc) overlap syndromes, RNAP—RNA polymerase.

**Table 2:** The most characteristic images of SSc in various organs.

Tissues and Internal Organs	Characteristic Picture
**Skin area**	Three phases in the development of cutaneous manifestations: ○ ‘Puffy finger phase’; ○ ‘Prolonged fibrotic phase’; ○ ‘Skin-softening phase’; Loss of elasticity of the skin; Strong feeling of tension; Thickening and induration; Skin calcifications
**Microvascular area**	The presence of giant capillaries (with an apical diameter ≥ 50 µm); The presence of abnormal vessel morphology with a reduced number of capillaries
**Gastrointestinal tract area**	Microstomia and xerostomia; Esophageal dysphagia; Lower esophageal sphincter (LOS) abnormalities; Gastresophageal reflux disease (GERD); Barrett’s esophagus; Gastric antral vascular ectasia (GAVE); Chronic intestinal pseudo-obstruction (CIPO); Small intestinal bacterial overgrowth syndrome (SIBO)
**Pulmonary area**	Vascular endothelial damage and dysregulated inflammatory response and angiogenesis; Systemic scleroderma-associated interstitial lung disease (SSc-ILD); Pulmonary arterial hypertension (PAH)
**Cardiac area**	Fibrous lesions in the coronary arteries, pericardium and myocardium; Myocarditis; Coronary artery disease; Myocardial fibrosis; Conduction system abnormalities; Valvular regurgitation; Heart failure; Pericardial and/or endocardial disease.
**Kidney area**	Damage to the renal glomeruli and impaired glomerular filtration; Scleroderma renal crisis (SRC): ○ Active and chronic thrombotic microangiopathy (TMA); ○ Malignant hypertension; ○ Rapidly progressive renal failure.

LOS—lower esophageal sphincter, GERD—gastresophageal reflux disease, GAVE—gastric antral vascular ectasia, CIPO—chronic intestinal pseudo-obstruction, SIBO - small intestinal bacterial overgrowth syndrome, SSc-ILD—systemic scleroderma-associated interstitial lung disease, PAH—pulmonary arterial hypertension, SRC—scleroderma renal crisis, TMA—thrombotic microangiopathy.

**Table 3:** Antibodies detected during the diagnosis of systemic sclerosis.

The Presence in the Patient’s Serum of Antibodies Against:	
**Topoisomerase I (anti-Scl 70)**	Significant skin involvement by fibrotic lesions; Greater probability of severe organ changes, especially SSc-ILD.
**Polymerase III (anti-RNAP III)**	Significant skin involvement by fibrotic lesions; More frequent renal complications, gastric vascular ectasias and neoplastic lesions.
**Centromere (anti-CENP-B, -A, -C or -D)**	Moderate degree of skin fibrosis; Slower organ involvement; Negatively correlation with the occurrence of neoplastic lesions.
**U11/U12 RNP (anti-RNPC3)**	Higher risk of severe GIT manifestations; Higher risk of neoplastic lesions.
**Fibrillarin (anti-U3 RNP)**	Higher risk of PAH and other cardiac complications.
**Th/To (anti-Th/To)**	Higher risk of PAH; Higher risk of SSc-ILD.
**Endothelial cells, the PDGF receptor and endothelin receptors**	Faster progressive fibrotic changes.
**The large RNA subunit Pol I (RPA194)**	Reduced risk of cancer.
**Neutrophil cytoplasm (ANCA)**	Higher risk of SSc-ILD with other pulmonary complications; Renal complications; Increased mortality.

anti-Scl 70—antibodies against topoisomerase I, anti-RNAP III - antibodies against RNA polymerase III, anti-CENP-B, -A, -C or –D - antibodies against centromeres of type -B, -A, -C or –D, anti-RNPC3—antibodies against RNA-binding region (U11/U12 RNP) containing 3, anti-Th/To—antibodies against a protein component shared by RNase P and RNase MRP, PDGF—platelet-derived growth factor, Pol I—polymerase I, RPA194—antibodies against the large RNA subunit polimerase I, ANCA—anti-neutrophil cytoplasmic antibodies, SSc-ILD—systemic scleroderma-associated interstitial lung disease, GIT—gastrointestinal tract, PAH—pulmonary arterial hypertension.

## Data Availability

The data used in the study are not sensitive data.
